# Ventricular tachycardia observed during cesarean section in a patient without structural cardiac disease

**DOI:** 10.1186/s40981-015-0019-0

**Published:** 2015-12-29

**Authors:** Mika Nakanishi, Kaoru Masumo, Takako Oota, Takeshi Kato, Toshihiro Imanishi

**Affiliations:** Department of Anesthesia, Osakafu Saiseikai Noe Hospital, Fruichi 1-3-25, Joto-ku, Osaka City, Osaka Japan

**Keywords:** Ventricular tachycardia, Cesarean section

## Abstract

A 32-year-old gravida 2, para 1 woman without structural cardiac disease was scheduled for her second cesarean section under combined spinal and epidural anesthesia (CSEA). She had stable hemodynamics after delivery; however, 16 min after the application of uterotonics, ventricular tachycardia (VT) with a heart rate (HR) of 150 bpm appeared. VT lasted for <30 s, and her hemodynamics remained stable. Ventricular arrhythmia frequently appeared for 3 min, and the HR at sinus rhythm was approximately 90 bpm. After the discontinuation of oxytocin, VT did not reappear. A postoperative 12-lead electrocardiogram showed first-degree atrioventricular block, but echocardiography performed 2 days later did not reveal any structural abnormalities. Autonomic nervous imbalance induced by CSEA, ephedrine, and oxytocin, as well as ergometrine may cause intraoperative VT during cesarean section in patients without structural cardiac disease.

## Background

Ventricular tachycardia (VT) is often observed during anesthesia. Arrhythmias lasting >30 s (sustained VT) may cause unstable hemodynamics and require treatment. Although there are some reports documenting the presence of VT during cesarean section, in most of these cases, VT was caused by the patients’ cardiac disease [1–3]. We report the case of non-sustained VT without structural cardiac disease under combined spinal and epidural anesthesia (CSEA) during cesarean section.

## Case presentation

A 32-year-old gravida 2, para 1 woman, weighing 56 kg, was scheduled for a repeat cesarean section which was indicated because of a previous cesarean section 3 years back. The previous cesarean section was performed under intrathecal anesthesia with hyperbaric bupivacaine. Oxytocin (5 units) and methylergometrine maleate (0.2 mg) were administered intravenously as uterotonics after the birth. Anesthetic complications were not observed. She had also undergone resection of an ovarian cyst at 16 weeks of gestation under CSEA using hyperbaric bupivacaine and ropivacaine. Anesthetic complications were not observed, and the progress of pregnancy was uneventful. A preoperative examination conducted before the second cesarean section revealed no signs or symptoms of cardiovascular disease, and she was classified as American Society of Anesthesiologists (ASA) physical status class 1.

**Fig. 1:**
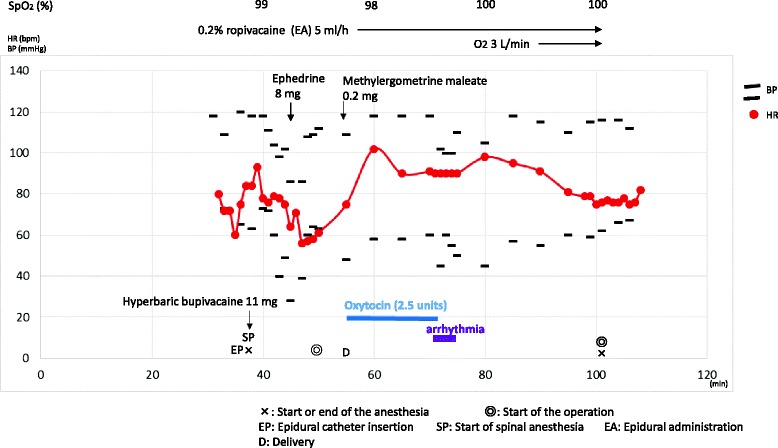
The anesthetic chart.

**Fig. 2:**
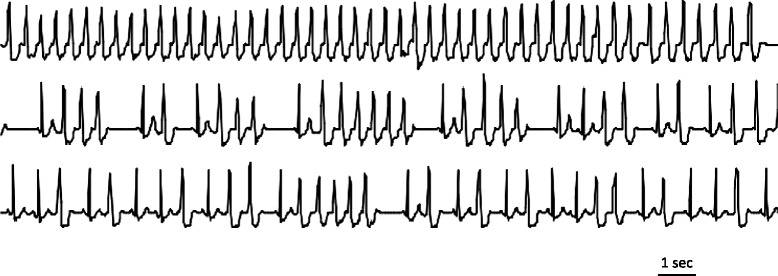
Intraoperative ventricular tachycardia (VT) appeared 16 min after the start of uterotonics in II-lead ECG. The longest VT lasted for 49 beats, and the heart rate (HR) was 150 bpm. Ventricular arrhythmia frequently appeared for 3 min

After an epidural catheter was inserted at the L2-3 level, hyperbaric bupivacaine (11 mg) was intrathecally administered at the L3-4 level. Eight minutes after the start of spinal anesthesia, ephedrine (8 mg) was intravenously injected because the blood pressure (BP) fell to 85/30 mmHg (Fig. 1). Her hemodynamics recovered immediately, and 10 min after the start of spinal anesthesia, epidural anesthesia (0.2 % ropivacaine 5 ml/h) was initiated. Seventeen minutes after the start of anesthesia, the child was delivered. After the birth, oxytocin (5 units) was mixed in a crystalloid solution (500 ml) and delivered intravenously over 30 min along with methylergometrine maleate (0.2 mg) injected over 10 min (Fig. 1). Sixteen minutes after the start of oxytocin and methylergometrine maleate, VT appeared. She complained of palpitations but was fully conscious. The longest period of ventricular rhythm lasted for 49 beats, and the heart rate (HR) was 150 bpm. Ventricular arrhythmia frequently appeared for 3 min (Fig. 2). BP during the arrhythmia was approximately 110/50 mmHg, and the HR at sinus rhythm was 90 bpm. Pulse oximetry showed oxygen saturation (SpO_2_) of 98 %. The anesthetic level was at the T4 dermatome. The injection of methylergometrine had been completed, but the intravenous oxytocin infusion was still ongoing. After the appearance of arrhythmia, the oxytocin infusion was stopped immediately and the arrhythmia disappeared. The total dose of oxytocin infused was 2.5 units. Temporary transcutaneous pacing pads combined with a defibrillator were prepared. However, she was fully conscious and needed to be sedated for their application. Before sedation was administered, the arrhythmia disappeared and the defibrillator and temporary pacing pads were not used. Anti-arrhythmic agents were not administered either, because ventricular arrhythmia did not reappear. Anesthetic duration was 1 h 3 min, and blood loss was 736 ml including amniotic fluid.

Ninety minutes after the appearance of VT, a 12-lead electrocardiogram (ECG) showed sinus rhythm at a rate of 60 bpm, a PR duration of 0.215 s, and no ST changes. In comparison with the preoperative PR duration, the postoperative PR duration was longer, suggesting first-degree atrioventricular (AV) block. Methylergometrine maleate (0.4 mg) was administered intravenously for 10 h after the surgery. During the following 24-h period, continuous ECG monitoring did not reveal any ventricular rhythm. A blood test performed 18 h later revealed normal electrolyte concentrations. Echocardiography performed 2 days later showed no structural cardiac abnormalities. The patient was discharged on postoperative day 8 without complications.

### Discussion

**Fig. 3:**
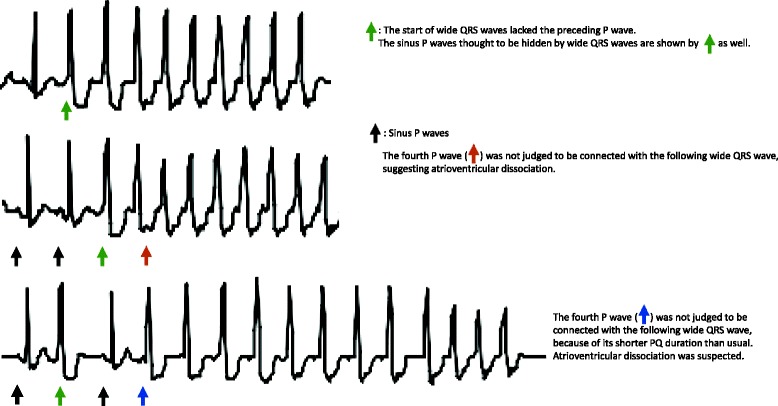
Wide QRS waves with no preceding P waves and atrioventricular dissociation strongly suggested that the arrhythmia was VT. *Green triangle*: The start of wide QRS waves lacked the preceding P wave. The sinus P waves thought to be hidden by wide QRS waves are shown by the *green arrow* as well. *Black arrow*: Sinus P waves. The fourth P wave (*orange arrow*) was not judged to be connected with the following wide QRS wave, suggesting atrioventricular dissociation. The fourth P wave (*blue arrow*) was not judged to be connected with the following wide QRS wave, because of its shorter PQ duration than usual. Atrioventricular dissociation was suspected

VT needs to be distinguished from supraventricular tachycardia (SVT) with aberrant conduction or bundle branch block. VT lacks the preceding P wave at the start of wide QRS waves, and atrioventricular dissociation is a differentiation point of VT from SVT. In this case, wide QRS waves with no preceding P waves and atrioventricular dissociation strongly suggested that the arrhythmia was VT (Fig. 3). VT observed in this case lasted for <30 s. Because of the short duration and the stable hemodynamics, the patient did not require any additional treatment.

Ergometrine is broadly used in coronary angiography and echocardiography for the ergonovine testing of coronary vasospasm [4, 5]. It may cause AV block and VT along with coronary vasospasm as a consequence of the ischemic impulse conduction system. In this case, ST changes were not observed. Postoperative infusion of ergometrine did not induce ventricular arrhythmia or ST changes. The rapid intraoperative infusion of ergometrine may have caused ventricular arrhythmia; however, the relationship between the coronary vasospasm and the arrhythmia in this case is unclear.

Oxytocin is also related to hemodynamics. Oxytocin-induced QTc prolongation is an indirect mechanism; however, oxytocin is known to affect the autonomic nervous nerve tone and the duration of cardiomyocyte repolarization and may induce arrhythmias [6]. In this case, the postoperative ECG revealed normal QTc duration, which contradicted the expected finding of oxytocin-induced prolongation.

Ephedrine is a sympathomimetic that has both direct (alpha and beta receptor agonist) and indirect (release of norepinephrine from presynaptic nerve terminals) mechanisms of action. It has a slow onset of action making it difficult to titrate the dose [7, 8]. The duration of action is approximately 60 min [8]. There is a case report of VT during general anesthesia, which is thought to have been caused by autonomic nervous imbalance because of a combination of ephedrine administration, insufficient anesthetic depth, neostigmine, and epidural block [9]. In this case, VT appeared 25 min after the administration of ephedrine. Ephedrine administration and arrhythmia could be related.

CSEA was performed in this case, but the total dose of local anesthetics was low, and local anesthetic-related toxicity was ruled out.

Oxytocin and ergometrine were administered during the previous cesarean section without complications. Using Naranjo’s adverse drug reaction probability scale [10], we obtained scores of 2 for ergometrine and 4 for oxytocin, which make them possible causes for the event. Autonomic nervous imbalance induced by CSEA, ephedrine, and oxytocin, as well as ergometrine, a vasoconstrictor, may have caused the arrhythmia in this case.

## Conclusions

Autonomic nervous imbalance induced by the combination of CSEA, ephedrine, and oxytocin, as well as ergometrine may cause VT in a patient without structural cardiac disease.

## Consent

The patient’s consent to publish this case report was obtained and documented.
